# Mundhöhlen- und Pharynxkarzinome: Inzidenz, Mortalität und Überleben in Deutschland

**DOI:** 10.1007/s00103-021-03368-z

**Published:** 2021-07-01

**Authors:** Lina Jansen, Julius Moratin, Annika Waldmann, Karim Zaoui, Bernd Holleczek, Alice Nennecke, Ron Pritzkuleit, Peter K. Plinkert, Jürgen Hoffmann, Volker Arndt

**Affiliations:** 1 Epidemiologisches Krebsregister Baden-Württemberg, Deutsches Krebsforschungszentrum (DKFZ), Im Neuenheimer Feld 581, 69120 Heidelberg, Deutschland; 2 Klinik und Poliklinik für Mund‑, Kiefer- und Gesichtschirurgie, Universitätsklinikum Heidelberg, Heidelberg, Deutschland; 3 Institut für Sozialmedizin und Epidemiologie, Universität zu Lübeck, Lübeck, Deutschland; 4 Hals-Nasen-Ohrenklinik, Universitätsklinikum Heidelberg, Heidelberg, Deutschland; 5 Krebsregister Saarland, Saarbrücken, Deutschland; 6 Hamburgisches Krebsregister, Hamburg, Deutschland; 7 Institut für Krebsepidemiologie e. V., Registerstelle des Krebsregisters Schleswig-Holstein, Lübeck, Deutschland; 8 Cancer Survivorship, Abt. klinische Epidemiologie und Alternsforschung, Deutsches Krebsforschungszentrum (DKFZ), Heidelberg, Deutschland

**Keywords:** Mundhöhlentumore, Pharynxtumore, Epidemiologie, Stadium, Prognose, Mouth neoplasms, Pharyngeal neoplasms, Epidemiology, Stage, Prognosis

## Abstract

**Hintergrund:**

Die Gruppe der Lippen‑, Mundhöhlen- und Pharynxkarzinome (ICD-10: C00–C14) beschreibt eine heterogene Gruppe bösartiger Tumoren, deren Inzidenz, Mortalität und Überleben sich nach Entität stark unterscheiden.

**Ziel der Arbeit:**

Diese Arbeit gibt eine detaillierte Übersicht epidemiologischer Maßzahlen für diese Tumorentitäten unter Berücksichtigung der Heterogenität bezüglich Alter, Geschlecht, Lokalisation und Stadium.

**Material und Methoden:**

Inzidenz- und Mortalitätsdaten für Deutschland für die Jahre 1999–2016 wurden aus der interaktiven Datenbank des Zentrums für Krebsregisterdaten (ZfKD) extrahiert. Alters- und Stadienverteilungen und altersstandardisiertes relatives 5‑Jahres-Überleben wurden auf dem gepoolten ZfKD-Datensatz (Diagnosejahre 1999–2017) berechnet.

**Ergebnisse:**

Im Jahr 2016 lagen die Inzidenz und Mortalität für alle Entitäten insgesamt bei 17,6 bzw. 7,0 pro 100.000 Männern und 6,5 bzw. 1,8 pro 100.000 Frauen. Das relative 5‑Jahres-Überleben für 2015–2017 lag bei 53 % bzw. 63 %. Es zeigten sich deutliche Unterschiede in der Überlebensrate und in der Alters- und Stadienverteilung zwischen den Entitäten. Im zeitlichen Verlauf zeigte sich ein Anstieg des Alters bei Diagnose, insbesondere bei Männern, aber keine Veränderung in der Stadienverteilung. Trotzdem stieg das relative 5‑Jahres-Überleben zwischen 1999–2002 und 2013–2017 von 45 % (Männer) bzw. 59 % (Frauen) auf 52 % bzw. 63 %.

**Diskussion:**

Die starke Heterogenität der untersuchten Tumoren verdeutlicht die Notwendigkeit einer nach Geschlechtern und Lokalisationen getrennten Betrachtung für eine aussagekräftige Interpretation der epidemiologischen Kennzahlen. Mit Ausbau der klinischen Krebsregistrierung werden in Zukunft zusätzliche Analysen unter Einbezug weiterer wichtiger klinischer Faktoren möglich sein.

**Zusatzmaterial online:**

Zusätzliche Informationen sind in der Online-Version dieses Artikels (10.1007/s00103-021-03368-z) enthalten.

## Abstract (en)

**Background:**

Lip, oral cavity, and pharynx cancers (ICD-10: C00–C14) describe a heterogeneous group of tumors with strong variations in incidence, mortality, and survival by entity.

**Objectives:**

This work provides a detailed overview of epidemiologic measures for these tumor entities, taking into account heterogeneity in age, sex, location, and stage.

**Material and Methods:**

Incidence and mortality data for Germany for the years 1999–2016 were extracted from the interactive database of the Center for Cancer Registry Data (ZfKD). Age and stage distributions and five-year relative survival were calculated on the pooled ZfKD data set (diagnosis years 1999–2017).

**Results:**

In 2016, overall incidence and mortality for all entities were 17.6 and 7.0 per 100,000 men and 6.5 and 1.8 per 100,000 women, respectively. The five-year relative survival in 2015–2017 was 53 and 63%, respectively. There were marked differences in survival as well as age and stage distributions between entities. Trend analyses showed an increase in age at diagnosis, particularly in male patients, and no change in stage distributions. However, five-year relative survival increased from 45% (men) and 59% (women) in 1999–2002 to 52% and 63% in 2013–2017.

**Conclusion:**

The marked heterogeneity of the studied tumors highlights the need to differentiate the analysis by sex and entity for meaningful interpretation of epidemiologic metrics. With the expansion of clinical cancer registration in Germany, additional analyses including other important clinical factors will be possible in the future.

## Einleitung

Die Gruppe der Lippen‑, Mundhöhlen- und Pharynxkarzinome (ICD-10: C00–C14, kurz: Mundhöhlen- und Pharynxkarzinome) beschreibt eine heterogene Gruppe bösartiger Tumorerkrankungen, die sich im Kopf-Hals-Bereich manifestieren. Die weitaus größte Gruppe bilden hierbei Plattenepithelkarzinome, die von den Schleimhäuten des oberen Aerodigestiv-Trakts, speziell denen der Mundhöhle, des Naso‑, Oro- und Hypopharynx, ausgehen. Weitere, kleinere Gruppen bilden außerdem bösartige Tumoren der Speicheldrüsen sowie die insgesamt selteneren Weichgewebstumoren oder Sarkome.

Weltweit bilden Plattenepithelkarzinome des Kopf-Hals-Bereichs (engl.: „head and neck squamous cell carcinoma“ – HNSCC) eine der 7 häufigsten Tumorentitäten, wobei deutliche regionale Unterschiede bezüglich Inzidenz und Geschlechterverteilung beobachtet werden [1]. Als wichtigste Risikofaktoren für eine Erkrankung galten lange Zeit der Konsum von Alkohol und Tabakwaren sowie in einigen Regionen, v. a. des südostasiatischen Raumes, das Kauen von Betelnüssen [2–4]. In den letzten Jahrzehnten nahm jedoch insbesondere bei Tumoren im Bereich des Oropharynx die Bedeutung viraler Infektionen, insbesondere mit sogenannten Hochrisikostämmen des Humanen Papillomavirus (HPV), stark zu [5, 6]. Die Gruppe der Patienten, die an einem HPV-assoziierten Oropharynxkarzinom leiden, unterscheidet sich typischerweise hinsichtlich Alter, Vorerkrankungen und Prognose von der Gruppe der an einem mit den klassischen Risikofaktoren assoziierten Karzinom Erkrankten, sodass der Virusnachweis zwischenzeitlich auch zu einer Unterscheidung in der aktuellen TNM-Klassifikation (englisch: tumor, node, metastasis) des Oropharynxkarzinoms geführt hat [7]. Die primäre Therapie von Plattenepithelkarzinomen des Kopf-Hals-Bereichs besteht in aller Regel in der chirurgischen Resektion mit adäquater Rekonstruktion sowie ggf. in einer adjuvanten Radio- oder Radiochemotherapie. Bei fortgeschrittenen Befunden oder nicht operationsfähigen Patienten kann alternativ eine primäre Radiochemotherapie in kurativer Intention erfolgen. Rein medikamentöse Therapieformen werden aktuell ausschließlich in nicht kurativ behandelbaren Krankheitsstadien eingesetzt [8].

Tumoren der Speicheldrüsen bilden innerhalb der Kopf-Hals-Tumoren eine gesonderte heterogene Gruppe aus vielen einzelnen und zum Teil sehr seltenen Entitäten, die hinsichtlich ihrer Ätiologie eher nicht durch klassische Risikofaktoren erklärt werden können. Sie zeichnen sich je nach histologischem Subtyp und Differenzierungsgrad durch eine sehr unterschiedliche Aggressivität und Prognose aus. Wie bei den Plattenepithelkarzinomen besteht die Therapie in der Regel aus der primären chirurgischen Resektion mit oder ohne adjuvante Therapie oder alternativ und in Abhängigkeit von ihrer Tumorlokalisation und Größe aus der primären Radiotherapie für fortgeschrittene, nicht resektable Tumore.

Die Überlebensraten der beschriebenen Tumorentitäten unterscheiden sich je nach Entität, Lokalisation und Stadium stark, wobei für Kopf-Hals-Karzinome generell Langzeitüberlebensraten zwischen 40 % und 80 % angegeben werden [9, 10]. Die in einzelnen Studien berichteten Inzidenz- und Überlebensraten beziehen sich meist auf bestimmte Subgruppen von Tumoren oder Lokalisationen und bilden daher häufig nur einen ungenügenden Querschnitt der gesamten Patientenpopulation ab.

Ziel der vorliegenden Studie war daher eine detaillierte Auswertung von Mundhöhlen- und Pharynxkarzinomen hinsichtlich der Inzidenz- und Mortalitätsraten, der relativen Überlebenszeiten und der Alters- und Stadienverteilung. Ein Fokus lag hierbei auf geschlechtsspezifischen Analysen für einzelne Lokalisationsgruppen.

## Methoden

Den Berechnungen liegen 2 Datenquellen zugrunde. Angaben zur Inzidenz und Mortalität wurden aus der interaktiven Datenbankabfrage des Zentrums für Krebsregisterdaten (ZfKD) extrahiert [11], die derzeit Daten zu Patienten mit Diagnosejahren bis 2016 beinhaltet. Da aus der Datenbank keine (detaillierten) Angaben zu Stadienverteilungen und Überlebensschätzern extrahiert werden können, wurde für alle weiteren Berechnungen der gepoolte Datensatz des ZfKD mit Daten bis einschließlich Diagnosejahr 2017 verwendet.

Unabhängig von der Datenquelle wurden Frauen und Männer mit neudiagnostiziertem Mundhöhlen- oder Pharynxkarzinom (ICD-10: C00–C14) eingeschlossen.

### Fallzahlen und Raten der Inzidenz und Mortalität

Die Anzahl der Fälle mit Mundhöhlen- oder Pharynxkarzinomen und die altersstandardisierten Inzidenz- und Mortalitätsschätzer in Deutschland für 1999–2016 wurden aus der Datenbank des ZfKDs extrahiert. Die Fallzahlen und Inzidenzraten sind Schätzungen für Deutschland, bei denen für regionale Untererfassungen korrigiert wurde. Die Inzidenz und Mortalität wurden als Rate pro 100.000 Personen dargestellt. Für die Altersstandardisierung wurde der alte Europastandard genutzt [12].

### Alters- und Stadienverteilungen und Krebsüberleben

Diese Analysen basieren auf dem gepoolten epidemiologischen Krebsregisterdatensatz des ZfKD. Der Datensatz wurde auf Regionen beschränkt, die eine ausreichende Datenqualität für Überlebenszeitanalysen in den Jahren 1999 bis 2017 hatten. Als Qualitätsindikatoren für die Vollzähligkeit wurde der Anteil der Fälle, deren Registrierung alleinig auf Todesbescheinigung basiert („death certificate only“, DCO) für Krebs gesamt (ICD-10: C00–C97 ohne sonstige bösartige Neubildungen der Haut (C44)) betrachtet (Kriterium: < 15 %). Zur Beurteilung der Follow-up-Qualität wurde das 1‑Jahres-Überleben nach Stadium IV Pankreas- und Lungenkrebs zwischen einzelnen Diagnosejahren und Regionen verglichen. Es konnten Daten von 13 der 16 Bundesländer in die Analyse einbezogen werden, wobei für einige Regionen eine Beschränkung der Kalenderjahre oder Regionen nötig war (Onlinetabelle 1).

Eingeschlossen wurden Personen mit neudiagnostiziertem Mundhöhlen- oder Pharynxkarzinom zwischen 1999 und 2017. DCO-Fälle wurden ausgeschlossen. Des Weiteren wurde pro Person jeweils nur der erste Tumor entsprechend der in der Analyse genutzten ICD-10-Gruppierung (s. unten) eingeschlossen.

#### Variablen

Betrachtet wurden die Variablen Diagnosejahr, Tumordiagnose (in Form des ICD-10-Codes), Geschlecht, Alter bei Diagnose und das UICC-Stadium (Union-for-International-Cancer-Control-Stadium; gebildet aus den TNM-Angaben). Bezüglich der ICD-10-Codes wurde folgende Gruppierung gewählt: Lippe (C00), Mundhöhle (C02–C06), Speicheldrüse (C07–C08), Oropharynx (C01, C09–C10), Nasopharynx (C11), Hypopharynx (C12–C13), Sonstige (C14). Bei allen Analysen nach UICC-Stadium wurden nur Patienten mit einem Karzinom (entsprechend des Histologiecodes nach ICD-O-3) und einer Codierung nach der TNM-Klassifikation maligner Tumore, 7. Auflage (TNM 7) eingeschlossen [13]. Im Rahmen dieser Klassifikation sind einige ICD-Subgruppen abweichend zu der o. g. Gruppierung zugeordnet: Die ICD-10-Codes C05.1 und C05.2 werden dem Oropharynx statt der Mundhöhle zugeordnet. C10.4 und C14 haben keine UICC-Stadien. C10.1 wird den Larynxtumoren zugeordnet, wird aber hier nicht weiter betrachtet, weil die Fallzahl zu gering ist. Für die restlichen Codes bleibt die Einteilung gleich. Bei Patienten ohne registrierte Angaben zum Vorliegen von Fernmetastasen wurde angenommen, dass solche nicht vorlagen.

### Statistik

Die Alters- und Stadienverteilung und die gemeinsame Verteilung von Alter und Stadium wurden getrennt nach Geschlecht und ICD-10-Gruppen für die Diagnosejahre 2015–2017 dargestellt. Unterschiede in den Verteilungen zwischen Männern und Frauen und zwischen Altersgruppen wurden mit einem Chi-Quadrat-Test auf Signifikanz geprüft. Zusätzlich wurde der zeitliche Verlauf zwischen 1999 (Altersverteilung) bzw. 2010 (Stadienverteilung) und 2017 untersucht. Hierbei wurde sich auf Regionen beschränkt, die Daten ab 1999 bzw. 2010 bereitstellten (Onlinetabelle 1).

Das relative 5‑Jahres-Überleben wurde für 3‑Jahres-Perioden als Verhältnis des absoluten zum erwarteten Überleben geschätzt. Für Diagnosejahre mit mindestens 5 Jahren Follow-up (1999–2002 und 2003–2007) erfolgte die Schätzung mithilfe der traditionellen Kohortenmethode. Für aktuellere Perioden wurde der Periodenansatz genutzt [14, 15]. Das erwartete Überleben wurde mit der Ederer-II-Methode aus deutschlandweiten Sterbetafeln nach Alter, Geschlecht und Kalenderjahr geschätzt [16, 17]. Alle Schätzer wurden für Frauen und Männer separat gerechnet und nach dem International Cancer Survival Standard altersstandardisiert [18]. Bei Analysen über alle Altersgruppen wurde die Gewichtung mit 5 Altersgruppen durchgeführt. Bei altersspezifischen Analysen wurde innerhalb der Altersgruppen altersstandardisiert. Da nicht für alle Bundesländer Daten für den gesamten Zeitraum verfügbar waren, wurden Verlaufsanalysen auf Regionen mit Krebsregisterdaten ab 1999 beschränkt. Schätzer werden nur gezeigt, wenn der Standardfehler ≤ 5 % war.

Unterschiede in den Überlebensraten zwischen Gruppen wurden mithilfe der modellbasierten Periodenanalyse auf Signifikanz getestet. Hierbei wurde in einem Poisson-Modell, mit dem Logarithmus der Personenjahre unter Risiko als Offset, die Anzahl der Sterbefälle als Funktion des Alters bei Diagnose, dem Follow-up-Jahr und dem Geschlecht modelliert [19].

Alle Analysen erfolgten mit SAS Enterprise Guide 7.15 (SAS Institute Inc., Cary, NC, USA).

## Ergebnisse

### Aktuelle Schätzungen der Inzidenz, Mortalität und des relativen Überlebens

**Figure 1:**
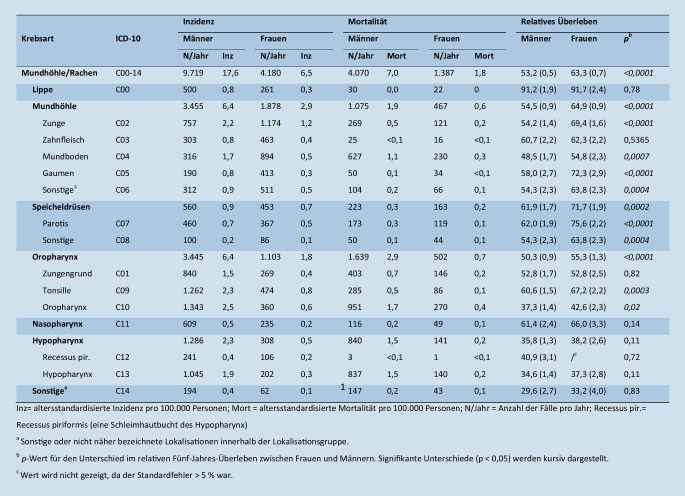

Im Jahr 2016 wurden geschätzt 13.789 Mundhöhlen- und Pharynxkarzinome in Deutschland neu diagnostiziert. Im selben Jahr starben 5457 Personen aufgrund dieser Erkrankungen (Abb. 1). Generell war sowohl die Inzidenz als auch die Mortalität bei Männern mit 17,6 bzw. 7,0 Fällen pro 100.000 Personen deutlich höher als bei Frauen (6,5 bzw. 1,8). Auch für die ICD-10-Gruppen war dieses Muster erkennbar, mit dem größten Geschlechterunterschied bei Oropharynxkarzinomen und dem geringsten Unterschied bei Speicheldrüsenkarzinomen. Die Krebsarten mit der höchsten Inzidenz innerhalb der Mundhöhlen- und Pharynxkarzinome waren bei Männern und Frauen Oropharynxkarzinome mit den Subgruppen Tonsillenkarzinome (C09: 2,3 und 0,8) und Oropharynxkarzinome (C10: 2,5 und 0,6) sowie Zungenkarzinome (C02: 2,2 und 1,2). Mit 1,7 bzw. 0,4 Fällen pro 100.000 Frauen bzw. Männern war auch die Mortalität bei der Subgruppe Oropharynxkarzinome (C10) am höchsten.

Für die Gesamtgruppe der Mundhöhlen- und Pharynxkarzinome lag das relative 5‑Jahres-Überleben bei 53 % bei Männern und 63 % bei Frauen. Frauen hatten eine signifikant höhere Überlebensrate bei Karzinomen der Mundhöhle (außer Zahnfleisch), der Speicheldrüsen und des Oropharynx (außer Zungengrund). Am größten waren die Geschlechterunterschiede bei Karzinomen der Zunge, des Gaumens und der Parotis (Ohrspeicheldrüse) mit einer 15, 14 bzw. 13 Prozentpunkte höheren relativen 5‑Jahres-Überlebensrate bei Frauen. Unabhängig vom Geschlecht gab es große Unterschiede im Überleben zwischen den Krebsarten innerhalb der Gruppe der Mundhöhlen- und Pharynxkarzinome mit den höchsten Überlebensraten bei Karzinomen der Lippe (Männer: 91 %, Frauen: 92 %) und den niedrigsten Überlebensraten bei sonstigen nicht näher bezeichneten Karzinomen (Männer: 30 %, Frauen: 33 %) und Hypopharynxkarzinomen (Männer: 35 %, Frauen: 37 %).

### Alters- und Stadienverteilungen

**Figure 2:**
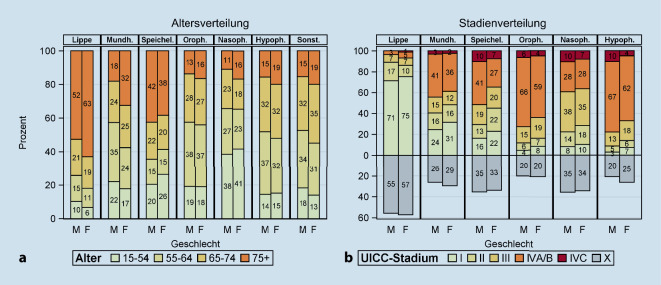

Der Anteil der Patienten und Patientinnen, die bei Diagnose 75 Jahre oder älter waren, war bei Karzinomen der Lippe mit 52 % bzw. 63 % am höchsten (Abb. 2a). Der Anteil war deutlich kleiner bei allen Pharynxkarzinomen (< 20 %). Signifikante Unterschiede in der Altersverteilung zwischen Männern und Frauen zeigten sich bei Karzinomen der Lippe (*p* = 0,0021), Mundhöhle (*p* < 0,0001) und des Oropharynx (*p* = 0,0062) mit einem größeren Anteil älterer Frauen.

Karzinome der Lippe hatten eine erheblich günstigere Stadienverteilung als die anderen Krebsarten mit einem Anteil von 71 % (Männer) bzw. 75 % (Frauen) Stadium-I-Fällen (3 % bzw. 6 % Stadium IV; Abb. 2b). Hierbei sollte aber berücksichtig werden, dass es insbesondere in dieser Gruppe einen hohen Anteil an fehlenden Stadienangaben gab (Männer: 55 %, Frauen: 57 %). Den größten Anteil an Stadium-IV-Fällen hatten Hypopharynxkarzinome mit 77 % (Männer) bzw. 66 % (Frauen), wobei aber nur 10 % bzw. 4 % der Fälle Metastasen bei Diagnose aufwiesen (Stadium IVc). Frauen hatten eine signifikant günstigere Stadienverteilung als Männer bei Karzinomen der Mundhöhle, der Speicheldrüse, des Oropharynx und des Hypopharynx (alle *p* < 0,0001).

**Figure 3:**
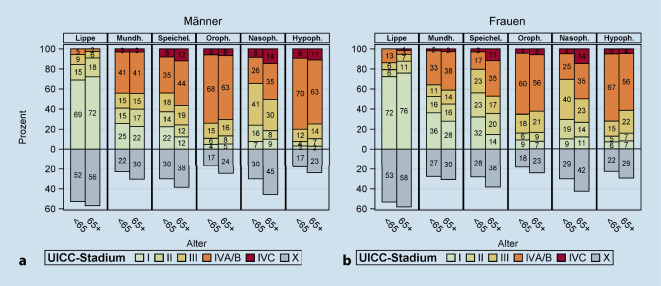

Abb. 3 zeigt die Stadienverteilung für Frauen und Männer getrennt nach Alter bei Diagnose (< 65 Jahre, ≥ 65 Jahre). Die Stadienverteilung war signifikant ungünstiger bei älteren als bei jüngeren Patienten bei Karzinomen der Speicheldrüse bei Männern (*p* = 0,0076) und Frauen (*p* < 0,0001) und bei Mundhöhlenkarzinomen bei Frauen (*p* = 0,0035). Bei Oropharynxkarzinomen hatten jüngere Patienten (aber nicht Frauen) eine signifikant ungünstigere Stadienverteilung (*p* = 0,0247). Bei den anderen Krebsarten zeigte sich kein signifikanter Zusammenhang mit dem Alter, wobei tendenziell der Anteil an Patienten mit fehlender Stadienangabe bei älteren Patienten höher war.

Zwischen 1999–2002 und 2013–2017 nahm der Anteil an älteren Patienten bei allen Krebsarten bei den Männern signifikant zu (Nasopharynx: *p* = 0,02, andere: *p* < 0,0001; Onlineabbildung 1a). Bei den Frauen stieg der Anteil weniger deutlich an, wobei sich die Altersverteilungen für 1999–2002 und 2013–2017 bei Lippen- (*p* = 0,0221), Mundhöhlen- und Oropharynx- (beide: *p* < 0,001) und Hypopharynxkarzinomen (*p* = 0,0005) signifikant unterschieden (Onlineabbildung 1b).

Die Stadienverteilung (nach Ausschluss der Fälle mit fehlenden Stadienangaben) änderte sich zwischen 2010/2011 und 2016/2017 bei allen Krebsarten nur geringfügig (Onlineabbildung 2). Signifikante Unterschiede wurden nur für Mundhöhlenkarzinome der Männer (*p* = 0,01) und Oropharynxtumoren der Frauen (*p* = 0,05) gefunden, wobei auch hier kein klarer Trend über die Diagnoseperioden erkennbar war.

### Aktuelles alters- und stadienspezifisches Überleben

**Figure 4:**
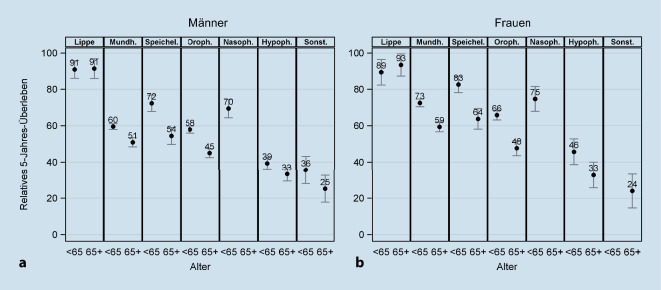

Die relativen 5‑Jahres-Überlebensraten in 2015–2017 waren sowohl bei den Frauen als auch bei den Männern für alle Krebsarten außer Lippenkarzinomen bei älteren Patienten (≥ 65 Jahre) signifikant geringer als bei jüngeren (alle *p* < 0,0001 außer bei „Sonstigen“: *p* = 0,0256 (Männer); Abb. 4). Am größten waren die Unterschiede bei Speicheldrüsen- (Männer: 18 Prozentpunkte, Frauen: 19 Prozentpunkte) und Oropharynxkarzinomen (13 bzw. 18 Prozentpunkte).

**Figure 5:**
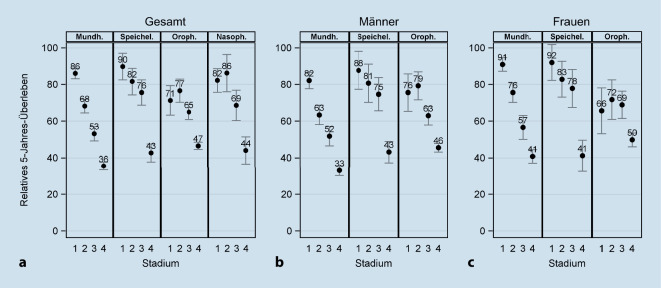

Stadienspezifisches relatives 5‑Jahres-Überleben in 2015–2017 konnte nur für Frauen und Männer gemeinsam für Nasopharynxkarzinome und geschlechtsspezifisch für Mundhöhlen‑, Speicheldrüsen- und Oropharynxkarzinome berechnet werden (Abb. 5). Die Überlebensrate sank bei Mundhöhlen- und Speicheldrüsenkarzinomen mit steigendem Stadium deutlich ab. So hatten Patienten mit Mundhöhlenkarzinomen im Stadium I eine Überlebenswahrscheinlichkeit von 82 % (Männer) bzw. 91 % (Frauen), wohingegen die Überlebensrate in Stadium IV 33 % bzw. 41 % betrug. Für Oropharynxkarzinome waren die Unterschiede zwischen den Stadien weniger deutlich ausgeprägt, wobei hier die Konfidenzintervalle sehr weit waren. Für Nasopharynxkarzinome war die Überlebensrate im Stadium I und II vergleichbar, gefolgt von einer deutlichen Verringerung im Stadium III und IV. Insgesamt war das stadienspezifische relative Überleben für Mundhöhlen- und Speicheldrüsenkarzinome tendenziell bei Frauen höher als bei Männern.

### Zeitliche Verläufe der Inzidenz, Mortalität und des relativen Überlebens

**Figure 6:**
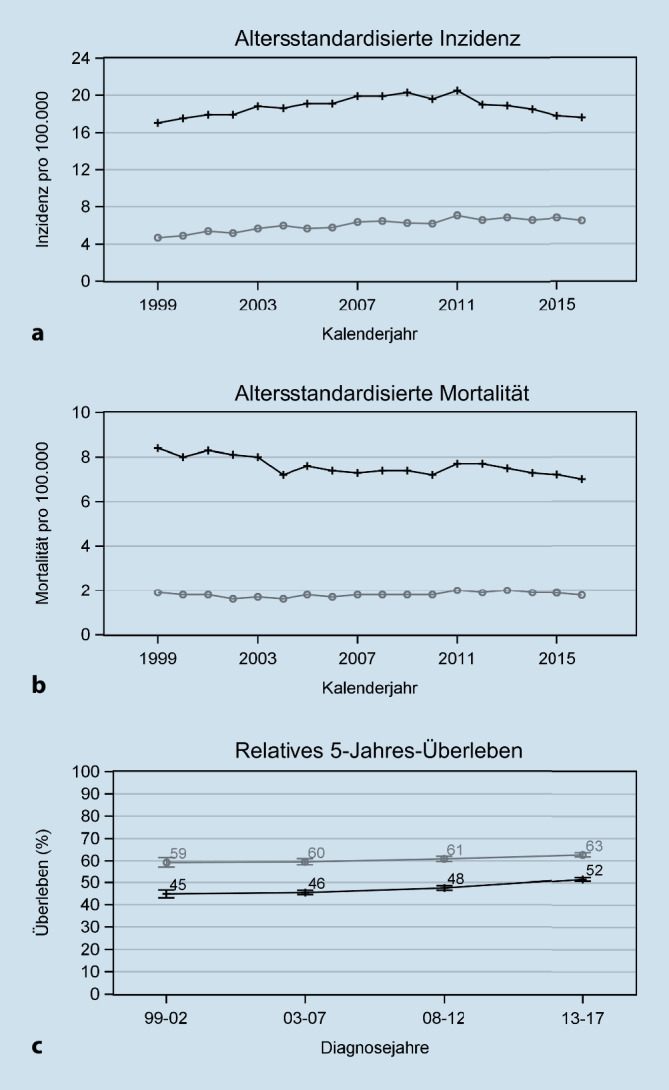

Abb. 6a, b zeigen die zeitlichen Verläufe der altersstandardisierten Inzidenz und Mortalität für Mundhöhlen- und Pharynxkarzinome in Deutschland nach Geschlecht zwischen 1999 und 2016. Entsprechende zeitliche Verläufe für einzelne Krebsarten sind in Onlineabbildung 3a bis b dargestellt.

Bei Männern stieg die Inzidenz pro 100.000 von 17,0 (1999) auf 20,5 (2011) und sank danach auf 17,6 (2016) ab. Dieser Rückgang der Inzidenz in den letzten Jahren war auf den Rückgang bei Mundhöhlen‑, Hypopharynx und Oropharynxkarzinomen zurückzuführen. Die Mortalität pro 100.000 für die Gesamtgruppe der Männer sank von 8,4 (1999) auf 7,0 (2017) ab, wobei aber schon in den Jahren 2004–2010 die Mortalität zeitweise bei 7,2 lag. Die krebsartspezifische Darstellung zeigte hier insgesamt eine Reduktion der Mortalität über die Jahre bei Mundhöhlen- und Hypopharynxkarzinomen, aber nicht bei Oropharynxkarzinomen.

Bei den Frauen stieg die Inzidenz für die Gesamtgruppe pro 100.000 von 4,7 in (1999) auf 7,1 (2011). In den darauffolgenden Jahren war sie etwas geringer mit 6,5 (2016). Der Zuwachs war hauptsächlich auf den Anstieg der Inzidenz von Mundhöhlen- und Oropharynxkarzinomen zurückzuführen. Die Mortalität schwankte über alle Jahre zwischen 1,6 und 2,0 pro 100.000 Frauen ohne erkennbaren Trend.

Trends für das altersstandardisierte relative 5‑Jahres-Überleben zwischen 1999–2002 und 2013–2017 sind für die Mundhöhlen- und Pharynxkarzinome insgesamt in Abb. 6c und für die einzelnen Krebsarten in Onlineabbildung 3e und f dargestellt. Bei Männern und Frauen stieg das relative Überleben über die Diagnosejahre von 45 % auf 52 % bzw. von 59 % auf 63 %. Lokalisationsspezifisch war aber nur ein Trend für Mundhöhlenkarzinome bei Männern mit einem Anstieg des Überlebens von 44 % auf 51 % erkennbar.

## Diskussion

Die Studie gibt einen aktuellen Überblick zur Epidemiologie bösartiger Tumoren des Mund- und Rachenbereichs in Deutschland. Insgesamt zeigte sich eine deutliche Tendenz zu höheren Inzidenz- und Mortalitätsraten bei Männern im Vergleich zu Frauen. Diese Beobachtung deckt sich größtenteils mit den Daten aus anderen Publikationen und wird in der Regel dadurch erklärt, dass die Prävalenz klassischer Risikofaktoren wie Alkohol- und Tabakkonsum bei Männern insgesamt stärker ausgeprägt ist [20]. Die Tatsache, dass die eher nicht mit diesen Risikofaktoren assoziierten Tumoren wie Speicheldrüsenkarzinome einen deutlich geringeren Unterschied in der Verteilung bei den Geschlechtern zeigen, stützt diese These.

Die Inzidenz- und Mortalitätsraten für die verschiedenen Typen von Mundhöhlen- und Pharynxkarzinomen unterscheiden sich weltweit beträchtlich. So gehören Mundhöhlenkarzinome zu den häufigsten malignen Tumoren im südlichen Asien [20]. Vergleicht man die hier ermittelten Inzidenz- und Mortalitätsraten mit den Daten anderer Länder, zeigt sich für Tumoren der Mundhöhle und Lippe in Deutschland eine etwa mit den Raten aus Westeuropa vergleichbare Inzidenz, die sich je nach Erhebung leicht über dem weltweiten Durchschnitt bewegt. Für Tumoren des Oropharynx zeigt sich eine global überdurchschnittliche Inzidenz, die in etwa den Werten anderer Länder mit hohem Entwicklungsstand entsprechen [21]. Hinsichtlich der Mortalität entsprechen die hier ermittelten Raten in etwa dem globalen Durchschnitt [20]. Während Tumoren der Lippe am ehesten in südlichen Ländern, insbesondere Australien und Neuseeland auftreten, bewegt sich die Inzidenz in Deutschland in etwa im weltweiten Durchschnitt [21].

Deutliche Unterschiede im relativen 5‑Jahres-Überleben zwischen den einzelnen Entitäten wurden mit Werten > 90 % für Lippenkarzinome und < 40 % für Hypopharynxkarzinome beobachtet. Diese Unterschiede erklären sich teilweise durch Unterschiede in der Stadienverteilung. Während Tumoren der Lippe in aller Regel früh erkannt und einer entsprechenden Therapie zugeführt werden können, entziehen sich andere Lokalisationen, wie beispielsweise der Hypopharynx, einer Inspektion durch den Patienten oder auch den Arzt. Dies führt zu einer späten Erkennung der Erkrankung, was mit einer erhöhten Rate an weit fortgeschrittenen Tumoren und somit häufig eingeschränkten Therapiemöglichkeiten und einer schlechteren Prognose einhergeht. Diese Tendenz zeigt sich eindeutig auch in den hier dargestellten Daten und verdeutlicht die Notwendigkeit der differenzierten Betrachtung der einzelnen Lokalisationen im Mund‑/Rachenbereich, die prognostisch eine ausgeprägte Heterogenität aufweisen.

Frauen hatten eine höhere relative 5‑Jahres-Überlebensrate als Männer nach Mundhöhlen‑, Speicheldrüsen- und Oropharynxkarzinomen, was sich mit den Vergleichsdaten aus internationalen Analysen und anderen Tumorentitäten deckt [20, 22, 23]. Für die global beobachteten Überlebensunterschiede existieren verschiedene Erklärungsansätze, die neben prognostisch relevanten hormonellen Unterschieden auch ein im Durchschnitt größeres Gesundheitsbewusstsein sowie eine generell gesündere Lebensweise bei Frauen beinhalten [22, 24]. Bei den meisten hier untersuchten Tumorentitäten war die Stadienverteilung für Frauen prognostisch günstiger, was mit den Daten aus anderen Untersuchungen übereinstimmt und die Unterschiede in der Überlebensrate zumindest teilweise erklären kann [25]. Diese Beobachtung ließe sich z. B. auch durch eine von einigen Autoren vermutete frühere Arztkonsultation durch Frauen erklären [22, 24, 26].

Hinsichtlich der Altersverteilung zeigt sich bei den meisten Entitäten eine Tendenz zu einem höheren Erkrankungsalter bei Frauen. Diese Beobachtung kann durch Unterschiede der Altersverteilung bei Männern und Frauen in der Bevölkerung erklärbar sein. Es könnte aber auch mit dem unterschiedlichen Risikoverhalten zusammenhängen, wonach Männer eher risikofaktorassoziierte Tumoren entwickeln und daher früher erkranken als Frauen. Das höchste Alter wurde bei Patienten mit Karzinomen der Lippe beobachtet. Hierbei handelt es sich typischerweise zum allergrößten Teil um Platten- und Basalzellkarzinome, die eine starke Assoziation mit einer chronischen Exposition von UV-Strahlung zeigen, wodurch sich das fortgeschrittene Erkrankungsalter erklären lässt. Nachfolgend zeigte sich das höchste Alter bei Patienten mit Speicheldrüsen-, gefolgt von Mundhöhlenkarzinomen. Das niedrigste Erkrankungsalter wiesen Patienten mit Karzinomen des Naso‑, Oro- und Hypopharynx auf. Das niedrige Alter bei Patienten mit Oro- und Nasopharynxkarzinomen ist zum Teil ätiologisch durch die Assoziation mit viralen Infektionen sowie im Fall von Nasopharynxkarzinomen durch genetische Risikofaktoren zu erklären. Der Anteil HPV-assoziierter Oropharynxkarzinome stieg in den letzten Jahren insbesondere in Nordamerika und Europa konstant an, wobei typischerweise jüngere Patienten betroffen sind [21, 27, 28]. Die hier beschriebenen Altersunterschiede, die sich mit der verfügbaren Literatur decken, verdeutlichen ebenfalls die Heterogenität der untersuchten Entitäten und die Notwendigkeit einer sorgfältigen Trennung in der Analyse.

In der vorliegenden Analyse zeigte sich insbesondere bei Männern eine Zunahme des Patientenalters bei Ersterkrankung für die meisten der untersuchten Tumorentitäten. Für die gesamte Gruppe der Kopf-Hals-Tumoren wird aufgrund des anhaltenden demografischen Wandels mit einem generellen Anstieg der Lebenserwartung sowie des Anteils älterer Menschen an der Gesamtbevölkerung eine Zunahme der Inzidenz um etwa 62 % im Vergleich zu den Inzidenzraten von 2012 geschätzt [21]. Dieser Wandel lässt mit einem zukünftig erhöhten Aufkommen an betagten und hochbetagten Patienten rechnen, die aufgrund höherer Raten an relevanten Begleiterkrankungen möglicherweise anspruchsvoller bezüglich einer adäquaten Tumortherapie sein werden.

Zur langfristigen Senkung der Inzidenz von Mundhöhlen- und Rachenkarzinomen haben mehrere Untersuchungen Vorsorgemaßnahmen wie die Reduktion von Risikofaktoren beispielsweise durch weitere Sanktionierung von Tabakkonsum oder Aufklärungskampagnen zur Vermeidung sexuell übertragbarer Erkrankungen (HPV-Infektionen) sowie Impfkampagnen als wichtigste Bausteine identifiziert [21]. Weiterhin können Maßnahmen zur Tumorfrüherkennung einen wesentlichen Beitrag dazu leisten, den Diagnosezeitpunkt in Richtung früherer Stadien zu verschieben und somit die Mortalitätsraten zu senken. Hierzu zählen sowohl Screeningprogramme als auch Informationskampagnen für behandelnde Ärzte und die Allgemeinbevölkerung zur Steigerung des Bewusstseins für die Existenz der genannten Entitäten [21, 25, 29, 30].

Im zeitlichen Verlauf zeigt sich eine tendenzielle Abnahme von Mundhöhlen- und Pharynxkarzinomen bei Männern nach 2011, während sich bei Frauen eine Zunahme der Inzidenz zwischen 1999 und 2011 gefolgt von einer weitestgehend stabilen Inzidenz feststellen ließ. Ein Erklärungsansatz hierfür liegt ebenfalls im Risikoverhalten und beschreibt eine tendenziell stärkere bzw. früher einsetzende Abnahme der Prävalenz der o. g. Risikofaktoren bei Männern im Vergleich zu Frauen [31, 32].

In der Gesamtgruppe der Mundhöhlen- und Pharynxkarzinome stieg das relative 5‑Jahres-Überleben zwischen 1999–2002 und 2013–2017 leicht an. Auch diese Tendenz zeigt sich in mehreren vergleichbaren Studien und spiegelt am ehesten der Etablierung neuer multimodaler Therapiekonzepte und verbesserter Nachsorgeprogramme wider [21, 33, 34].

Die Stärken der hier vorliegenden Untersuchung sind der umfassende Überblick zu relevanten epidemiologischen Kennzahlen für verschiedene Tumorentitäten der Mundhöhlen- und Pharynxkarzinome differenziert nach Geschlecht über einen größeren Zeitraum hinweg. Hierdurch wird neben einer Erhebung des aktuellen Status quo auch eine Analyse von Trends im entsprechenden Zeitraum ermöglicht. Als problematisch ist die erhebliche Anzahl an Fällen ohne dokumentiertes Tumorstadium zu benennen. Dies erschwert die Interpretation sowohl der Stadienverteilung als auch des stadienspezifischen Überlebens. Durch den Ausschluss der DCO-Fälle, für die kein Diagnosedatum vorliegt, kann von einer leichten Überschätzung der tatsächlichen Überlebensraten ausgegangen werden, da DCO-Fälle tendenziell kürzere Überlebenszeiten haben [35]. Die Tatsache, dass nur Daten von 13 Bundesländern eingeschlossen werden konnten und von einigen Ländern die jeweiligen Informationen nicht für den kompletten Zeitraum vorlagen, verringert die Repräsentativität des Datensatzes, da die Fälle und Raten für das gesamte Bundesgebiet somit näherungsweise aus den verfügbaren Daten geschätzt wurden. Die Beschränkung der verfügbaren Datensätze auf Basisdaten zur Diagnose lässt zudem keine Analyse weiterer relevanter klinischer Faktoren zu. Solche erweiterten Daten werden zukünftig durch die flächendeckende klinische Krebsregistrierung nach § 65c Sozialgesetzbuch, fünftes Buch (SGB V) verfügbar werden [36]. Eine hohe Datenqualität in der Erfassung der Diagnose, der Therapie und des Verlaufs ist dabei essenziell, um zukünftig noch bestehende Informationslücken zu schließen, bislang unterrepräsentierte Regionen einzuschließen und zu besserem Erkenntnisgewinn und einer Steigerung der Effektivität und Effizienz des Gesundheitssystems beizutragen.

Die vorliegende Studie gibt einen umfassenden Überblick zu verschiedenen Tumorentitäten innerhalb der Mundhöhlen- und Pharynxkarzinome in Deutschland. Hierbei fallen vor allem eine Zunahme des Patientenalters bei Erstdiagnose, insbesondere bei männlichen Patienten, und ein Anstieg der Überlebensrate im Zeitverlauf ohne Verbesserung der Stadienverteilung auf. Die starke Heterogenität der untersuchten Tumoren verdeutlicht die Notwendigkeit einer nach Geschlechtern und Entitäten getrennten Betrachtung für eine aussagekräftige Interpretation. Die Verbesserung der Qualität der flächendeckenden klinischen Krebsregistrierung und eine zentrale Koordinierung der Datenzusammenführung aus den klinischen Krebsregistern nach § 65c SGB V können zudem dazu beitragen, die daraus ableitbaren Erkenntnisse und Maßnahmen auch in Zukunft zu verbessern.

## Supplementary Information


