# Supplementary material for: Oral and pharyngeal cancer: incidence, mortality, and survival in Germany
Source: Bundesgesundheitsblatt Gesundheitsforschung Gesundheitsschutz. 2021 Jul 1;64(8):941–50. [Article in German] doi: 10.1007/s00103-021-03368-z (PMC8316202; doi:10.1007/s00103-021-03368-z)

Bundesgesundheitsblatt 8/2021

Elektronisches Zusatzmaterial zum Beitrag:

Mundhöhlen- und Pharynxkarzinome: Inzidenz, Mortalität und Überleben in Deutschland

Lina Jansen[1]*, Julius Moratin[2]*, Annika Waldmann[3], Karim Zaoui[4], Bernd Holleczek[5], Alice Nennecke[6], Ron Pritzkuleit[7], Peter K. Plinkert[4], Jürgen Hoffmann[2]*, Volker Arndt[1,8]*
* geteilte Erst-/Letztautorenschaft

[1] Epidemiologisches Krebsregister Baden-Württemberg, Deutsches Krebsforschungszentrum (DKFZ), Heidelberg, Deutschland
[2] Klinik und Poliklinik für Mund-, Kiefer- und Gesichtschirurgie, Universitätsklinikum Heidelberg, Heidelberg, Deutschland
[3] Institut für Sozialmedizin und Epidemiologie, Universität zu Lübeck, Lübeck, Deutschland
[4] Hals-Nasen-Ohrenklinik, Universitätsklinikum Heidelberg, Heidelberg, Deutschland
[5] Krebsregister Saarland, Saarbrücken, Deutschland
[6] Hamburgisches Krebsregister, Hamburg, Deutschland
[7] Institut für Krebsepidemiologie e.V., Registerstelle des Krebsregisters Schleswig-Holstein, Lübeck, Deutschland
[8] Cancer Survivorship, Abt. klinische Epidemiologie und Alternsforschung, Deutsches Krebsforschungszentrum (DKFZ), Heidelberg, Deutschland

Korrespondenzadresse:
Dr. sc. hum. Lina Jansen
Epidemiologisches Krebsregister Baden-Württemberg,
Deutsches Krebsforschungszentrum (DKFZ)
Im Neuenheimer Feld 581
69120 Heidelberg
l.jansen@dkfz.de

Inhalt:

Onlinetabelle 1 Übersicht über die eingeschlossenen Krebsregister im Diagnosezeitraum 1999-2017

Onlineabbildung 1 Trends in der Altersverteilung zwischen 1999-2002 und 2013-2017 für Subgruppen von Mundhöhlen- und Pharynxkarzinomen bei Männern (a) und Frauen (b)

Onlineabbildung 2 Trends in der Stadienverteilung für Subgruppen von Mundhöhlen- und Pharynxkarzinomen bei Männern (a) und Frauen (b) zwischen 2010-2011 und 2016-2017

Onlineabbildung 3 Trends in altersstandardisierter Inzidenz (a/b), Mortalität (c/d) und altersstandardisiertem relativem Fünf-Jahres-Überleben (95 % Konfidenzintervall) (e/f) für Subgruppen von Mund- und Pharynxkarzinomen bei Männern (links) und Frauen (rechts).

Onlinetabelle 1 Übersicht über die eingeschlossenen Krebsregister im Diagnosezeitraum 1999-2017

	Einschluss Inzidenz und Mortalität	Einschluss Überlebenszeitanalysen			
Register	Bevölkerung (Mio. in 2016)	Diagnosejahre	Vitalstatus Follow-up bis	Krebs Gesamt DCO-Fälle[a]	C00-C14 Fälle[b]
Schleswig-Holstein	2,88	1999-2017	31.12.2017	10%	8471
Hamburg	1,81	1999-2017	31.12.2017	4%	5608
Niedersachsen	7,95	2003-2017	31.12.2017	8%	17044
Bremen	0,68	1999-2017	31.12.2017	7%	2198
Nordrhein-Westfalen	17,87	1999-2017[c]	31.12.2017	10%	27529
Hessen	6,21	/[d]	/	/	/
Rheinland-Pfalz	4,07	1999-2017	31.12.2017	2%	9779
Baden-Württemberg	10,95	2010-2017	31.12.2017	8%	11529
Freistaat Bayern	12,93	2002-2017	31.12.2017	7%	28347
Saarland	1,00	1999-2017	31.12.2017	8%	4020
Berlin	3,57	/[d]	/	/	/
Brandenburg	2,49	1999-2015	31.12.2015	11%	7317
Mecklenburg-Vorpommern	1,61	1999-2015	31.12.2015	10%	5982
Freistaat Sachsen	4,08	1999-2015	31.12.2015	7%	11884
Sachsen-Anhalt	2,24	/[d]	/	/	/
Freistaat Thüringen	2,16	1999-2015	31.12.2015	9%	5670
Gesamt	82,52	1999-2015/17		9%	145378

[a] Anteil der Fälle der alleinig auf der Todesbescheinigung basiert (death certificate only, DCO) an allen Krebsfällen (C00-C97 ohne sonstige bösartige Neubildungen der Haut (C44) 2015-2017 (in den östlichen Bundesländern 2015). [b] Nach Ausschluss von DCO Fällen. [c] Beschränkung auf den Regierungsbezirk Münster für die Diagnosejahre 1999-2009. [d] Ausschluss nach der Prüfung der Datenqualität

Onlineabbildung 1 Trends in der Altersverteilung zwischen 1999-2002 und 2013-2017 für Subgruppen von Mundhöhlen- und Pharynxkarzinomen bei Männern (a) und Frauen (b)

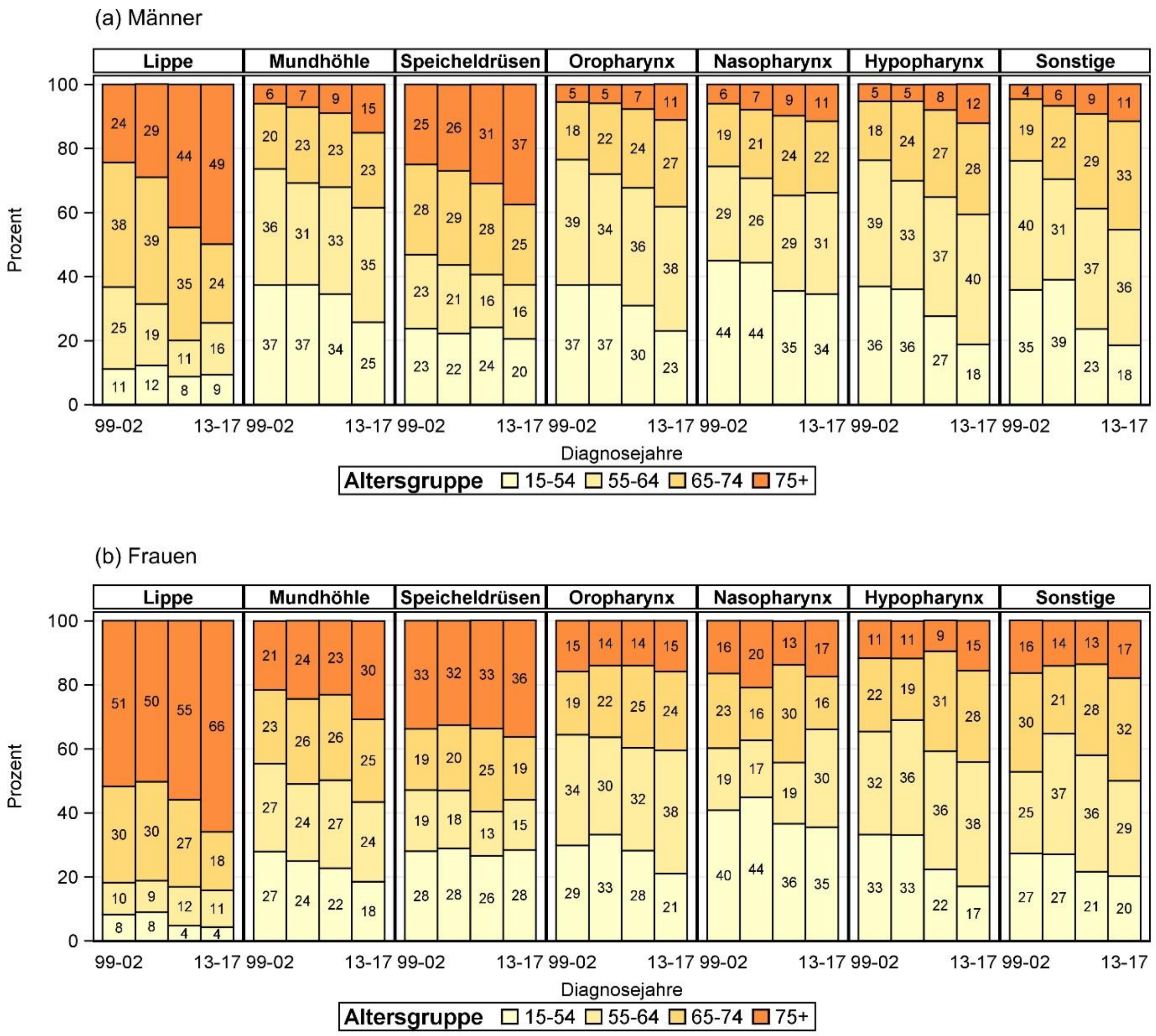

Onlineabbildung 2 Trends in der Stadienverteilung für Subgruppen von Mundhöhlen- und Pharynxkarzinomen bei Männern (a) und Frauen (b) zwischen 2010-2011 und 2016-2017

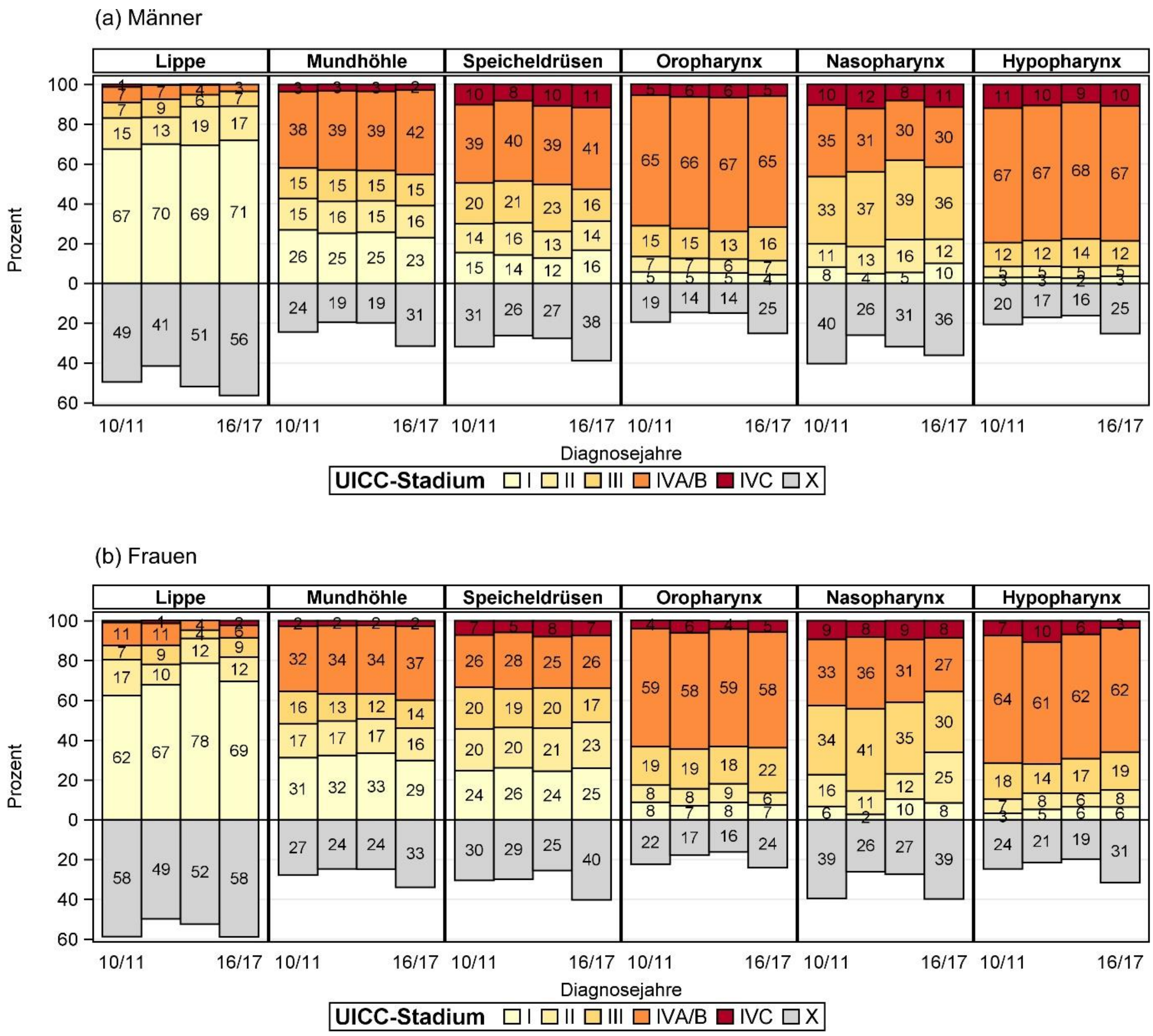

Onlineabbildung 3 Trends in altersstandardisierter Inzidenz (a/b), Mortalität (c/d) und altersstandardisiertem relativem Fünf-Jahres-Überleben (95 % Konfidenzintervall) (e/f) für Subgruppen von Mund- und Pharynxkarzinomen bei Männern (links) und Frauen (rechts). Relative Überlebensraten mit Standardfehlern größer 5 % werden nicht dargestellt.

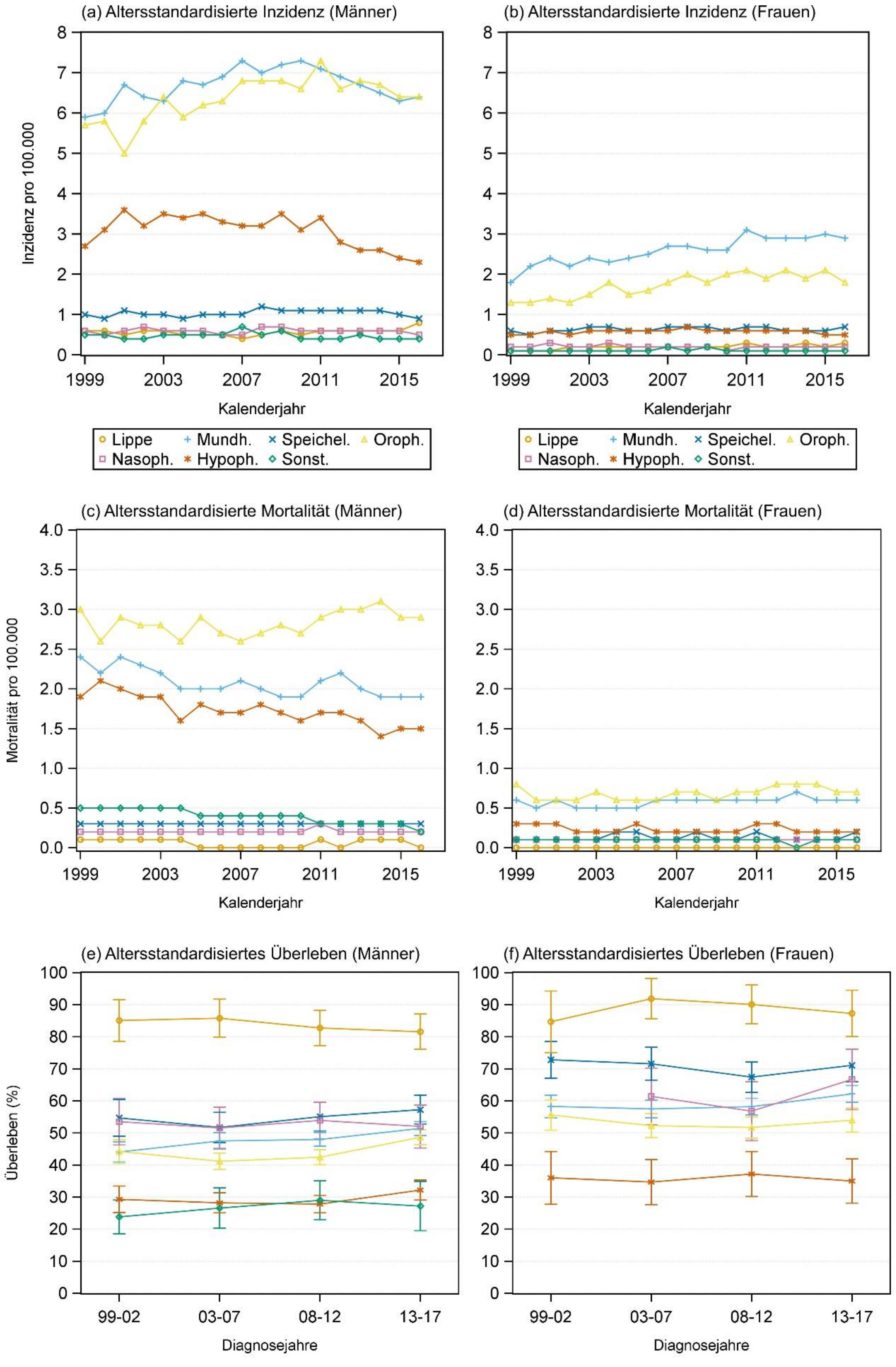

Supplement: Supplementary file 1 [file 103_2021_3368_MOESM1_ESM.pdf]
